# Dynamic evolution of canine parvovirus in Thailand

**DOI:** 10.14202/vetworld.2020.245-255

**Published:** 2020-02-10

**Authors:** N. Inthong, S. Kaewmongkol, N. Meekhanon, K. Sirinarumitr, T. Sirinarumitr

**Affiliations:** 1 Center for Agricultural Biotechnology, Kasetsart University, Kamphaeng Sean Campus, Nakhon Pathom 73140, Thailand; 2 Center of Excellence on Agricultural Biotechnology: (AG-BIO/PERDO-CHE), Bangkok 10900, Thailand; 3 Department of Veterinary Technology, Faculty of Veterinary Technology, Kasetsart University, 50 Ngamwongwan Road, Chatuchak 10900, Thailand; 4 Department of Companion Animal Clinical Sciences, Faculty of Veterinary Medicine, Kasetsart University, 50 Ngamwongwan Road, Chatuchak 10900, Thailand; 5 Department of Pathology, Faculty of Veterinary Medicine, Kasetsart University, 50 Ngamwongwan Road, Chatuchak, Bangkok 10900, Thailand

**Keywords:** canine parvoviruses, diversity, Thailand, *VP2* gene

## Abstract

**Background and Aim:**

According to the previous study, the circulating canine parvovirus (CPV) in Thailand is 2a and 2b. Nowadays, CPV mutants, including CPV-2c, have been identified in many parts of the world. This study aimed to investigate the genetic diversity of the circulating CPV in Thailand.

**Materials and Methods:**

Eighty-five CPV-positive fecal samples were obtained from dogs with either acute hemorrhagic diarrhea or diarrhea. The complete *VP2* gene of these samples was amplified using *VP2* specific primers and polymerase chain reaction (PCR). The obtained full-length *VP2* sequences were analyzed and a phylogenetic tree was constructed.

**Results:**

Sixty and 25 CPV-positive fecal samples were collected in 2010 and 2018, respectively. Thirty-four samples were new CPV-2a and 31 samples were new CPV-2b due to amino acids substitution at position 297 (Ser-Ala). In 2018, 5 new CPV-2a, 19 CPV-2c, and 1 feline panleukopenia virus (FPV) were found, but no new CPV-2b was detected. Moreover, most of the CPV in this study had amino acids mutations at positions 324 and 440. The phylogenetic construction demonstrated the close relationship between the current new CPV-2a with the previous CPV-2a reported from Thailand, China, Uruguay, Vietnam, Singapore, and India. Interestingly, the current new CPV-2b in this study was not closely related to the previous CPV-2b reported in Thailand. The CPV-2c in this study was closer to Asian CPV-2c and further from either European or South America CPV-2c. Interestingly, FPV was identified in a diarrhea dog.

**Conclusion:**

The evolution of CPV in Thailand is very dynamic. Thus, it is important to monitor for CPV mutants and especially the clinical signs relating to these mutants to conduct surveillance for the emergence of new highly pathogenic CPV in the future.

## Introduction

Canine parvovirus (CPV) is one of the most common viruses in domestic dogs. It causes acute hemorrhagic gastroenteritis, leukopenia, nausea, diarrhea, and sometimes fatal myocarditis in young puppies [1]. CPV belongs to the family *Parvoviridae*, subfamily *Parvovirinae*, and genus *Parvovirus*. It is a non-enveloped, icosahedral, linearized, and single-stranded DNA virus. The genome of CPV is approximately 5.2 kb in length. The virus encodes two nonstructural proteins (*NS1* and *NS2*) and three structural proteins (*VP1*, *VP2*, and *VP3*). The *VP2* capsid protein is the main capsid protein and plays an important role in the determination of the antigenicity and host range of CPV [2]. CPV type 2 (CPV-2) was first identified in the USA in 1978 and was found to have spread worldwide in domestic and wild canid populations [3]. CPV is genetically close to feline panleukopenia virus (FPV); however, CPV has at least seven amino acid differences from FPV which determine the canine or feline host range, such as amino acid positions 80 (Lys-Arg), 93 (Lys-Asn), 103 (Val-Ala), 232 (Val-Ile), 323 (Asp-Asn), 564 (Asn-Ser), and 568 (Ala-Gly) [4].

The original CPV-2 was replaced worldwide by CPV-2a and CPV-2b in 1985. CPV-2a and CPV-2b have some nucleotide changes at the *VP2* gene compared to the original CPV-2. There are six amino acid differences at residues 87 (Met-Leu), 101 (Ile-Thr), 300 (Ala-Gly), 305 (Asp-Tyr), 375 (Asn-Asp), and 555 (Val-Ile) between CPV-2 and CPV-2a and six amino acid differences at residues 87 (Met-Leu), 101 (Ile-Thr), 300 (Ala-Gly), 305 (Asp-Tyr), 375 (Asn-Asp), and 426 (Asn-Asp) between CPV-2 and CPV-2b. The differences between CPV-2a and CPV-2b are the substitution of two amino acids in the *VP2* capsid protein, namely, Asn-426 in 2a (Asp-426 in 2b) and Ile-555 in 2a (Val-555 in 2b). Recently, the emergence of new CPV-2a and CPV-2b has been reported having an amino acid mutation at position 297 (Ser-Ala). Moreover, the new CPV-2a has a mutation at amino acid position 555 that changes isoleucine back to valine [4-8]. CPV-2c is a new CPV strain that has a glutamate substitution at the 426 residues of the *VP2* protein [9-11]. Recently, CPV-2c has been detected in Argentina [12], Australia [13], Italy [10], Laos [14], Spain [6], Taiwan [15], and Uruguay [16]. According to the information above, CPV has a high mutation rate and has been dynamically evolving in many parts of the world. There is a growing concern about the severity and effectiveness of vaccine regarding the new mutant or genotype of CPV.

Molecular surveillance may be used as a tool for the detection of the new mutant, prediction of disease severity and providing the important data for the development of the better vaccine or the better diagnostic test in the future. Moreover, the knowledge of the current genotype of the CPV in Thailand is limited. This study aimed to investigate the current genotype of CPV circulating in Thailand and to determine the existence of CPV-2c and other CPV strains.

## Materials and Methods

### Ethical approval

This study was approved by the Animal Ethics Committee of the Faculty of Veterinary Medicine, Kasetsart University, Thailand (ACKU62-VET-007).

### Samples

Eighty-five fecal samples were used in this study based on a positive result for CPV according to routine polymerase chain reaction (PCR) testing. In 2010 and 2018, 60 and 25 positive samples, respectively, were collected (Table-1). These fecal samples were collected from dogs that displayed either acute hemorrhagic diarrhea or diarrhea and nausea at the Veterinary Teaching Hospital, Kasetsart University, Rattanatibeth Referral Animal Hospital, Bangkok, Thailand, and the Amphawa Pet Hospital, Samut Songkhram, Thailand. These dogs were either vaccinated or unvaccinated and were aged from 1 month to 5 years. The fecal samples were stored at −80°C until used for DNA extraction.

**Table-1:** Age of parvovirus-infected dogs, vaccination, year of sample collection, GenBank accession number, genotype of CPV, and amino acid at important position (NR=no report).

No.	Sample	Amino acid position																	CPV type	Previous vaccination	Age	GenBank accession number	Year of collection

		80	87	93	103	232	267	297	300	305	323	324	370	426	440	555	564	568					
1	FPV-VT-2020	K	M	N	V	V	F	S	A	D	D	Y	Q	N	T	V	N	A	FPV	NR	1 years	MN270937	2018
2	CPV-VT-1	R	L	N	A	I	Y	A	G	Y	N	I	Q	N	A	V	S	G	New 2a	No	NR	KP715658	2010
3	CPV-VT-7	R	L	N	A	I	Y	A	G	Y	N	I	Q	N	A	V	S	G	New 2a	No	3 M	KP715659	2010
4	CPV-VT-13	R	L	N	A	I	F	A	G	Y	N	I	Q	N	T	V	S	G	New 2a	No	3 M	KP715660	2010
5	CPV-VT-14	R	L	N	A	I	Y	A	G	Y	N	I	Q	N	A	V	S	G	New 2a	No	2 M	KP715661	2010
6	CPV-VT-30	R	L	N	A	I	F	A	G	Y	N	I	Q	N	T	V	S	G	New 2a	Yes	3 M	KP715662	2010
7	CPV-VT-37	R	L	N	A	I	F	A	G	Y	N	I	Q	N	A	V	S	G	New 2a	Yes	2 M	KP715663	2010
8	CPV-VT-39	R	L	N	A	I	Y	A	G	Y	N	I	Q	N	A	V	S	G	New 2a	No	5 M	KP715664	2010
9	CPV-VT-40	R	L	N	A	I	Y	A	G	Y	N	I	Q	N	A	V	S	G	New 2a	No	2 M	KP715665	2010
10	CPV-VT-42	R	L	N	A	I	Y	A	G	Y	N	I	Q	N	A	V	S	G	New 2a	No	1 M	KP715666	2010
11	CPV-VT-45	R	L	N	A	I	Y	A	G	Y	N	I	Q	N	A	V	S	G	New 2a	Yes	2 M	KP715667	2010
12	CPV-VT-51	R	L	N	A	I	Y	A	G	Y	N	I	Q	N	A	V	S	G	New 2a	Yes	6 M	KP715668	2010
13	CPV-VT-58	R	L	N	A	I	Y	A	G	Y	N	I	Q	N	A	V	S	G	New 2a	No	2 M	KP715669	2010
14	CPV-VT-61	R	L	N	A	I	Y	A	G	Y	N	I	Q	N	A	V	S	G	New 2a	No	3 M	KP715670	2010
15	CPV-VT-62	R	L	N	A	I	Y	A	G	Y	N	I	Q	N	A	V	S	G	New 2a	No	>3 M	KP715671	2010
16	CPV-VT-71	R	L	N	A	I	Y	A	G	Y	N	I	Q	N	A	V	S	G	New 2a	Yes	5 M	KP715672	2010
17	CPV-VT-81	R	L	N	A	I	Y	A	G	Y	N	I	Q	N	A	V	S	G	New 2a	No	4 M	KP715673	2010
18	CPV-VT-82	R	L	N	A	I	Y	A	G	Y	N	I	Q	N	A	V	S	G	New 2a	No	2 M	KP715674	2010
19	CPV-VT-83	R	L	N	A	I	Y	A	G	Y	N	I	Q	N	A	V	S	G	New 2a	No	3 M	KP715675	2010
20	CPV-VT-87	R	L	N	A	I	Y	A	G	Y	N	I	Q	N	A	V	S	G	New 2a	No	2 M	KP715676	2010
21	CPV-VT-88	R	L	N	A	I	Y	A	G	Y	N	I	Q	N	A	V	S	G	New 2a	NR	3 M	KP715677	2010
22	CPV-VT-89	R	L	N	A	I	Y	A	G	Y	N	I	Q	N	A	V	S	G	New 2a	No	1.5 M	KP715678	2010
23	CPV-VT-92	R	L	N	A	I	Y	A	G	Y	N	I	Q	N	A	V	S	G	New 2a	No	3 M	KP715679	2010
24	CPV-VT-93	R	L	N	A	I	Y	A	G	Y	N	I	Q	N	A	V	S	G	New 2a	No	3 M	KP715680	2010
25	CPV-VT-97	R	L	N	A	I	Y	A	G	Y	N	I	Q	N	A	V	S	G	New 2a	No	3 M	KP715681	2010
26	CPV-VT-103	R	L	N	A	I	Y	A	G	Y	N	I	Q	N	A	V	S	G	New 2a	No	>3 M	KP715682	2010
27	CPV-VT-109	R	L	N	A	I	Y	A	G	Y	N	I	Q	N	A	V	S	G	New 2a	NR	NR	KP715683	2010
28	CPV-VT-115	R	L	N	A	I	Y	A	G	Y	N	I	Q	N	A	V	S	G	New 2a	NR	NR	KP715684	2010
29	CPV-VT-138	R	L	N	A	I	Y	A	G	Y	N	I	Q	N	A	V	S	G	New 2a	NR	3 M	KP715685	2010
30	CPV-VT-139	R	L	N	A	I	Y	A	G	Y	N	I	Q	N	A	V	S	G	New 2a	NR	3.5 M	KP715686	2010
31	CPV-VT-0561	R	L	N	A	I	Y	A	G	Y	N	I	Q	N	A	V	S	G	New 2a	NR	NR	MN270938	2018
32	CPV-VT-1377	R	L	N	A	I	Y	A	G	Y	N	I	Q	N	A	V	S	G	New 2a	NR	NR	MN270939	2018
33	CPV-VT-2097	R	L	N	A	I	Y	A	G	Y	N	I	Q	N	A	V	S	G	New 2a	NR	2 M	MN270940	2018
34	CPV-VT-2098	R	L	N	A	I	Y	A	G	Y	N	I	Q	N	A	V	S	G	New 2a	NR	2 M	MN270941	2018
35	CPV-VT-2961	R	L	N	A	I	Y	A	G	Y	N	I	Q	N	A	V	S	G	New 2a	NR	1 M	MN270942	2018
36	CPV-VT-3	R	L	N	A	I	Y	A	G	Y	N	I	Q	D	T	V	S	G	New 2b	No	2 years	KP715687	2010
37	CPV-VT-12	R	L	N	A	I	Y	A	G	Y	N	I	Q	D	T	V	S	G	New 2b	Yes	7 M	KP715688	2010
38	CPV-VT-18	R	L	N	A	I	Y	A	G	Y	N	I	Q	D	T	V	S	G	New 2b	No	2 M	KP715689	2010
39	CPV-VT-28	R	L	N	A	I	Y	A	G	Y	N	I	Q	D	T	V	S	G	New 2b	Yes	3 M	KP715690	2010
40	CPV-VT-43	R	L	N	A	I	Y	A	G	Y	N	I	Q	D	T	V	S	G	New 2b	NR	1 years	KP715691	2010
41	CPV-VT-49	R	L	N	A	I	Y	A	G	Y	N	I	Q	D	T	V	S	G	New 2b	Yes	4 M	KP715692	2010
42	CPV-VT-53	R	L	N	A	I	Y	A	G	Y	N	I	Q	D	A	V	S	G	New 2b	No	2 M	KP715693	2010
43	CPV-VT-54	R	L	N	A	I	Y	A	G	Y	N	I	Q	D	T	V	S	G	New 2b	No	2 M	KP715694	2010
44	CPV-VT-56	R	L	N	A	I	Y	A	G	Y	N	Y	Q	D	T	V	S	G	New 2b	No	5 M	KP715695	2010
45	CPV-VT-65	R	L	N	A	I	Y	A	G	Y	N	I	Q	D	T	V	S	G	New 2b	No	NR	KP715696	2010
46	CPV-VT-66	R	L	N	A	I	Y	A	G	Y	N	I	Q	D	T	V	S	G	New 2b	No	2 years	KP715697	2010
47	CPV-VT-68	R	L	N	A	I	Y	A	G	Y	N	I	Q	D	T	V	S	G	New 2b	No	4 M	KP715698	2010
48	CPV-VT-74	R	L	N	A	I	Y	A	G	Y	N	I	Q	D	T	V	S	G	New 2b	No	NR	KP715699	2010
49	CPV-VT-75	R	L	N	A	I	Y	A	G	Y	N	I	Q	D	T	V	S	G	New 2b	No	2 M	KP715700	2010
50	CPV-VT-80	R	L	N	A	I	Y	A	G	Y	N	I	Q	D	T	V	S	G	New 2b	No	4 M	KP715701	2010
51	CPV-VT-84	R	L	N	A	I	Y	A	G	Y	N	I	Q	D	T	V	S	G	New 2b	No	4 M	KP715702	2010
52	CPV-VT-86	R	L	N	A	I	Y	A	G	Y	N	I	Q	D	T	V	S	G	New 2b	No	5 M	KP715703	2010
53	CPV-VT-90	R	L	N	A	I	Y	A	G	Y	N	I	Q	D	T	V	S	G	New 2b	Yes	6 M	KP715704	2010
54	CPV-VT-99	R	L	N	A	I	Y	A	G	Y	N	I	Q	D	T	V	S	G	New 2b	No	5 M	KP715705	2010
55	CPV-VT-101	R	L	N	A	I	Y	A	G	Y	N	I	Q	D	T	V	S	G	New 2b	No	1 M	KP715706	2010
56	CPV-VT-106	R	L	N	A	I	Y	A	G	Y	N	I	Q	D	T	V	S	G	New 2b	NR	NR	KP715707	2010
57	CPV-VT-108	R	L	N	A	I	Y	A	G	Y	N	I	Q	D	A	V	S	G	New 2b	NR	NR	KP715708	2010
58	CPV-VT-114	R	L	N	A	I	Y	A	G	Y	N	I	Q	D	T	V	S	G	New 2b	NR	NR	KP715709	2010
59	CPV-VT-120	R	L	N	A	I	Y	A	G	Y	N	I	Q	D	T	V	S	G	New 2b	No	3 M	KP715710	2010
60	CPV-VT-121	R	L	N	A	I	Y	A	G	Y	N	I	Q	D	T	V	S	G	New 2b	No	2 M	KP715711	2010
61	CPV-VT-123	R	L	N	A	I	Y	A	G	Y	N	I	Q	D	T	V	S	G	New 2b	No	8 M	KP715712	2010
62	CPV-VT-129	R	L	N	A	I	Y	A	G	Y	N	I	Q	D	T	V	S	G	New 2b	No	1.5 years	KP715713	2010
63	CPV-VT-135	R	L	N	A	I	Y	A	G	Y	N	I	Q	D	T	V	S	G	New 2b	No	NR	KP715714	2010
64	CPV-VT-142	R	L	N	A	I	Y	A	G	Y	N	I	Q	D	T	V	S	G	New 2b	NR	1 M	KP715715	2010
65	CPV-VT-143	R	L	N	A	I	Y	A	G	Y	N	I	Q	D	T	V	S	G	New 2b	No	4 M	KP715716	2010
66	CPV-VT-148	R	L	N	A	I	Y	A	G	Y	N	I	Q	D	A	V	S	G	New 2b	NR	2 M	KP715717	2010
67	CPV-VT-0861	R	L	N	A	I	Y	A	G	Y	N	I	R	E	T	V	S	G	2c	NR	1 M	MN270943	2018
68	CPV-VT-0937	R	L	N	A	I	Y	A	G	Y	N	I	R	E	T	V	S	G	2c	NR	NR	MN270944	2018
69	CPV-VT-1161	R	L	N	A	I	Y	A	G	Y	N	I	R	E	T	V	S	G	2c	NR	11 M	MN270945	2018
70	CPV-VT-1261	R	L	N	A	I	Y	A	G	Y	N	I	R	E	T	V	S	G	2c	NR	6 M	MN270946	2018
71	CPV-VT-1361	R	L	N	A	I	Y	A	G	Y	N	I	R	E	T	V	S	G	2c	NR	4 M	MN270947	2018
72	CPV-VT-1373	R	L	N	A	I	Y	A	G	Y	N	I	R	E	T	V	S	G	2c	NR	NR	MN270948	2018
73	CPV-VT-1374	R	L	N	A	I	Y	A	G	Y	N	I	R	E	T	V	S	G	2c	NR	NR	MN270949	2018
74	CPV-VT-1375	R	L	N	A	I	Y	A	G	Y	N	I	R	E	T	V	S	G	2c	NR	NR	MN270950	2018
75	CPV-VT-1383	R	L	N	A	I	Y	A	G	Y	N	I	R	E	T	V	S	G	2c	NR	NR	MN270951	2018
76	CPV-VT-1661	R	L	N	A	I	Y	A	G	Y	N	I	R	E	T	V	S	G	2c	NR	1 years	MN270952	2018
77	CPV-VT-2016	R	L	N	A	I	Y	A	G	Y	N	I	R	E	T	V	S	G	2c	NR	2 M	MN270953	2018
78	CPV-VT-2018	R	L	N	A	I	Y	A	G	Y	N	I	R	E	T	V	S	G	2c	NR	3 M	MN270954	2018
79	CPV-VT-2019	R	L	N	A	I	Y	A	G	Y	N	I	R	E	T	V	S	G	2c	NR	3 years	MN270955	2018
80	CPV-VT-2021	R	L	N	A	I	Y	A	G	Y	N	I	R	E	T	V	S	G	2c	NR	7 M	MN270956	2018
81	CPV-VT-2388	R	L	N	A	I	Y	A	G	Y	N	I	R	E	T	V	S	G	2c	NR	3 M	MN270957	2018
82	CPV-VT-2389	R	L	N	A	I	Y	A	G	Y	N	I	R	E	T	V	S	G	2c	NR	5 M	MN270958	2018
83	CPV-VT-2391	R	L	N	A	I	Y	A	G	Y	N	I	R	E	T	V	S	G	2c	NR	2.5 years	MN270959	2018
84	CPV-VT-2461	R	L	N	A	I	Y	A	G	Y	N	I	R	E	T	V	S	G	2c	NR	3 M	MN270960	2018
85	CPV-VT-3761	R	L	N	A	I	Y	A	G	Y	N	I	R	E	T	V	S	G	2c	NR	21 days	MN270961	2018

CPV=Canine parvovirus, FPV=Feline panleukopenia virus

### DNA extraction and PCR

DNA was extracted from the fecal samples using the acid guanidinium thiocyanate-phenol-chloroform extraction method. A set of primers was designed to amplify the whole *VP2* gene: F (5’-ATG AGT GAT GGA GCA GTT CA) and R (5’-TTA ATA TAA TTT TCT AGG TGC TAG TTG). The PCR mixture (25 µl) was composed of 1× buffer (20 mM Tris-HCl [pH 8.4], 50 mM KCl_2_), 0.2 mM dNTPs, 2.5 mM MgCl_2_, 100 pmol of each of the forward and reverse primers, 1 unit Taq DNA polymerase (Invitrogen, Carlsbad, CA, USA), and 2.5 µl of DNA template to give a total volume of 25 µl. After an initial denaturing at 94°C for 5 min, the amplification was performed using 35 cycles at 94°C for 40 s, annealing at 50°C for 40 s, and extension at 72°C for 90 s, and a final extension at 72°C for 10 min. The expected PCR products were 1755 bps in size. The PCR products were analyzed using 1% agarose gel electrophoresis at 100 V for 30 min and visualized under ultraviolet illumination. The PCR products were purified using an UltraClean^®^15 DNA Purification Kit (MO BIO Laboratories, Inc., Carlsbad, CA, USA) and cloned into plasmid pGEM-T Easy (Promega Corporation, Madison, WI, USA). The sequences of the cloned full-length *VP2* were determined at First BASE Laboratories Sdn Bhd, Selangor, Malaysia.

### Analysis and phylogenetic construction of full-length VP2 gene of CPVs

The nucleotide sequences were translated into amino acid and multiple alignments of the amino acid sequences using the Bioedit biological sequence alignment editor computer package (version 7.1.3; Ibis Biosciences, Carlsbad, CA, USA). The phylogenetic analysis was constructed from the amino acid sequences of all 85 samples in this study and other full-length *VP2* sequences obtained from the GenBank database (Table-2) using the MEGA program (version 7.0, The Biodesign Institute, Tempe, AZ, USA) with the neighbor-joining method and running 1000 replicates in the bootstrap. Bayesian phylogenetic analysis was also performed for more extensive amino acids analysis to analyze the selective pressures on certain amino acids using mixed model analysis. The phylogenetic tree was created by MrBayes version 3.2.6 (https://nbisweden.github.io/MrBayes/download.html) [17]. The tree was viewed using FigTree software version 1.4.3 (http://tree.bio.ed.ac.uk/software/figtree/).

**Table-2:** GenBank accession numbers of CPV used in phylogenetic tree construction.

Order	Origin	GenBank accession number	Year of collection	Genetic type
1	China	MF467224	2015	2a
2	China	FJ435343	2008	2a
3	China	FJ435345	2008	2a
4	China	GU380304	2009	2a
5	China	GU569936	2008	2a
6	France	DQ026002	-	2a
7	India	KX469433	2015	2a
8	Italy	FJ005259	2008	2a
9	Singapore	KY083098	2014	2a
10	Thailand	GQ379047	2009	2a
11	Thailand	GQ379045	2009	2a
12	Thailand	GQ379046	2009	2a
13	Uruguay	KM457139	2011	2a
14	Uruguay	JF906788	2010	2a
15	Vietnam	LC214970	2013	2a
16	China	GU569937	2002	2b
17	China	KF482468	2009	2b
18	China	GU569938	2002	2b
19	China	GU569944	2002	2b
20	China	JQ743891	2010	2b
21	Italy	FJ005263	2005	2b
22	Thailand	FJ869123	2008	2b
23	Thailand	FJ869124	2008	2b
24	Vietnam	AB120724	2013	2b
25	USA	JX475261	2010	2b
26	Argentina	KM236569	2013	2c
27	China	KT162005	2014	2c
28	China	KT162016	2014	2c
29	China	KP260509	2014	2c
30	China	KY937641	2016	2c
31	Croatia	KP859576	2014	2c
32	Ecuador	KF149984	2012	2c
33	Germany	FJ005202	1997	2c
34	Germany	FJ005204	1999	2c
35	Greece	GQ865518	2008	2c
36	Indonesia	LC216905	2013	2c
37	Italy	HQ025913	2010	2c
38	Singapore	KY083092	2014	2c
39	Uruguay	KM457112	2008	2c
40	-	M74849	-	2b
41	-	M24003	-	2a
42	-	M38245	-	2
43	-	FJ405225	-	FPV

CPV=Canine parvovirus, FPV=Feline panleukopenia virus

## Results

Thirty-nine and nine samples out of 85 positive samples were from non-vaccinated and vaccinated dogs, respectively. The other 37 positive samples did not have a history of vaccination. The youngest CPV-infected dog was aged 21 days, and the oldest was 3 years. The youngest age of positive vaccinated dogs was 2 months; however, the individual histories of booster vaccination for these positive, vaccinated dogs were not available (Table-1).

**Figure-1:**
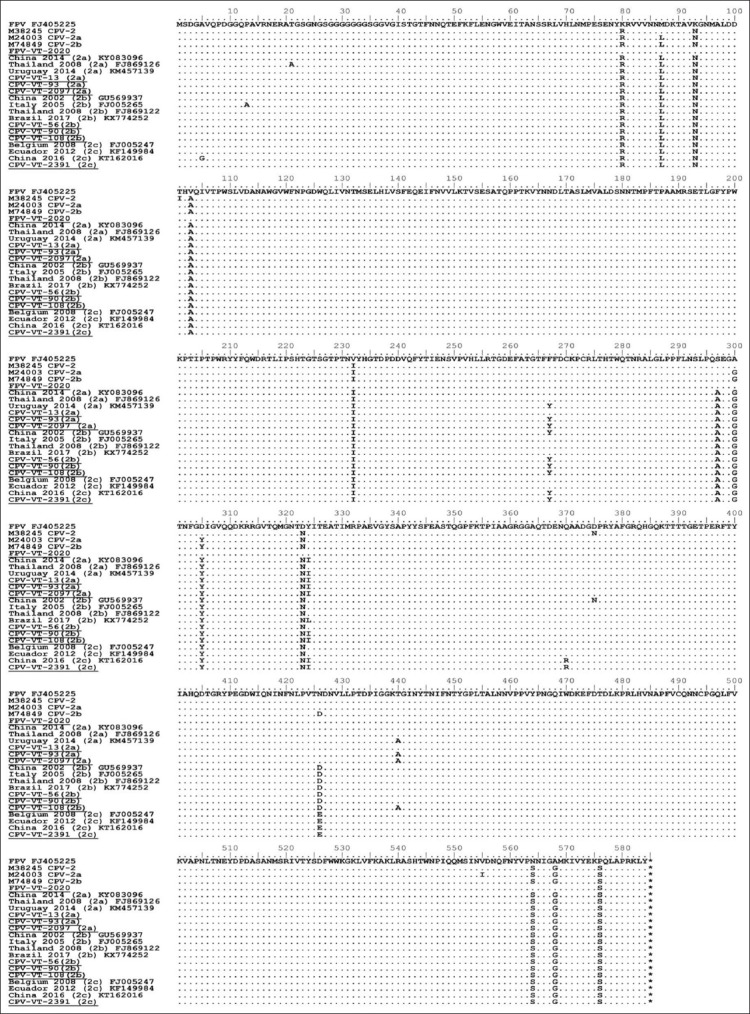
Amino acid comparison of *VP2* gene of CPV-2a: CPV-VT13 (T440), CPV-VT93 (A440), CPV-VT2097, CPV-2b: CPV-VT56 (324Y), CPV-VT90 (440T), and CPV-VT108 (440A), CPV-2c: CPV-VT2391, FPV: FPV-VT2020 in this study, with reference strains FPV (FJ405225), CPV-2 (M38245), CPV-2a (M24003) and CPV-2b (M74849) and other isolates from other parts of the world. CPV=Canine parvovirus, FPV=Feline panleukopenia virus.

**Figure-2:**
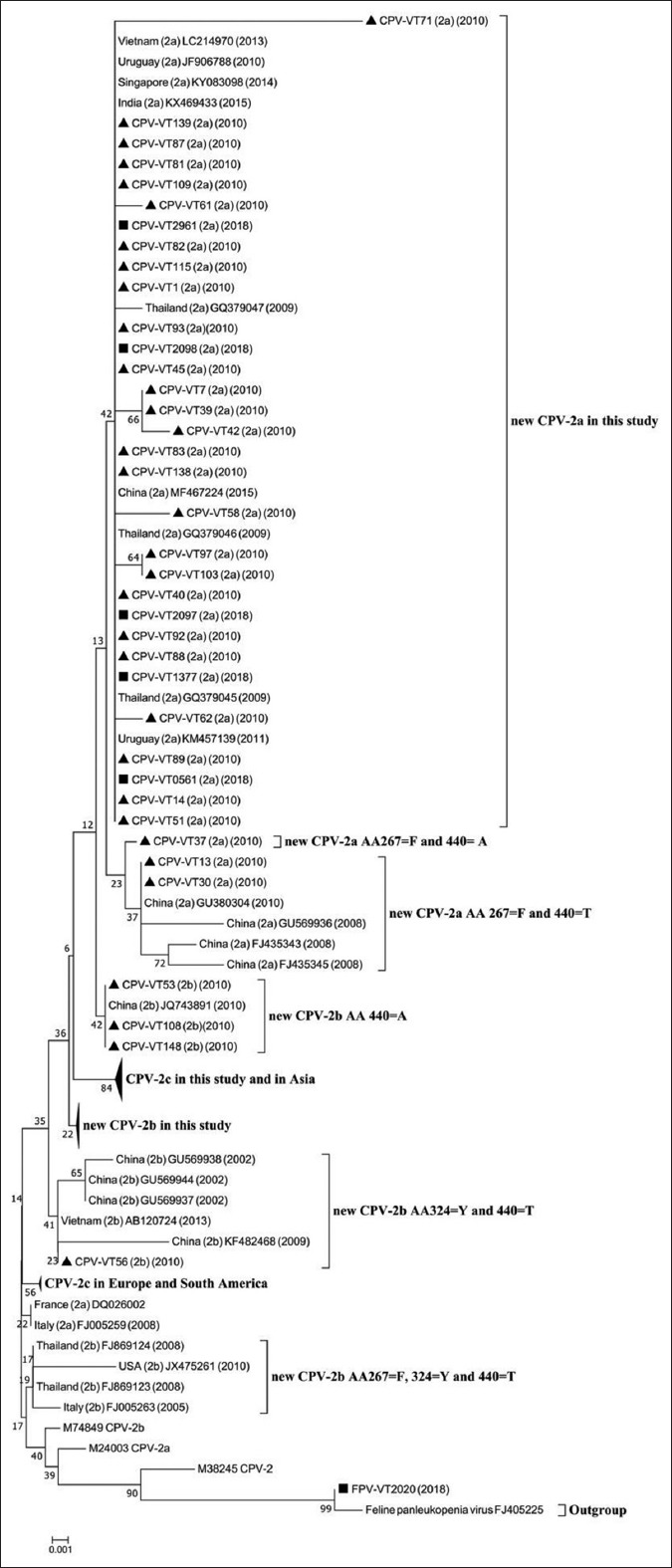
Phylogenetic tree constructed from 80 amino acid sequences of the *VP2* gene of canine parvovirus (CPV) and feline panleukopenia virus (FPV) in this study and other CPV and FPV sequences obtained from GenBank database using the neighbor-joining method and bootstrap analysis performed with 1000 trials. Drawn using MEGA version. **■**=Samples collected in 2018 and **▲**=Samples collected in 2010.

**Figure-3:**
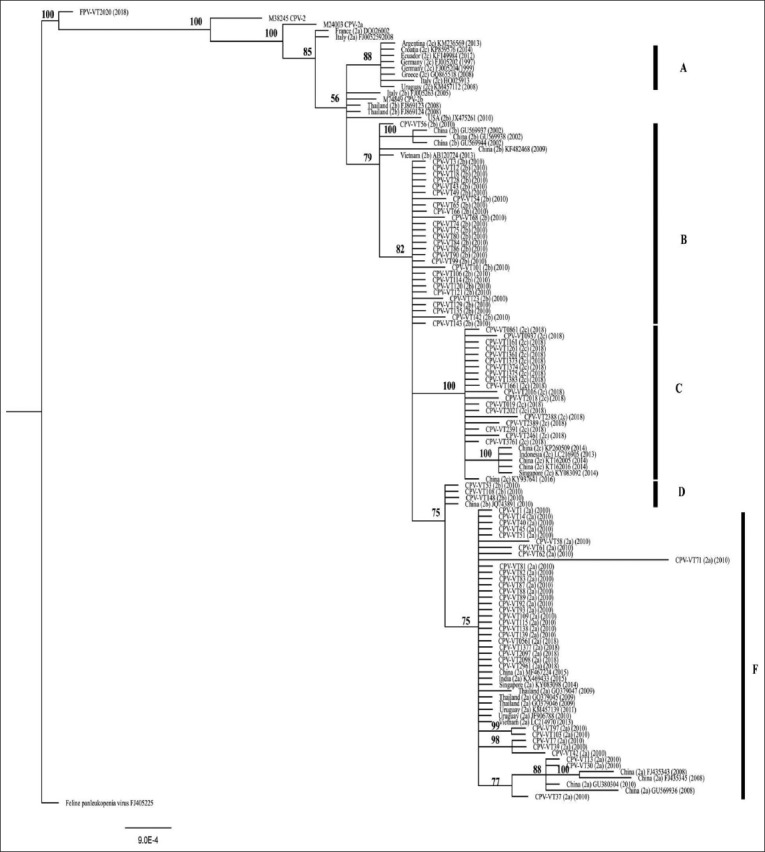
Phylogenetic tree constructed from 80 amino acids of the *VP2* gene of canine parvovirus (CPV) and feline panleukopenia virus (FPV) in this study and other CPV and FPV sequences obtained from GenBank database using MrBayes version 3.2.6 with Markov chain Monte Carlo, with 5 million generations. Node values (in percentages) indicate posterior clade probabilities. Vertical bars indicate clades of CPV in each group, A=CPV-2c in Europe and South America, B=new CPV-2b in this study, C=CPV-2c in this study, and Asia, D=new CPV-2b that has alanine at amino acid position 440, F=new CPV-2a in this study.

For the 2010 data, the amino acid sequences analysis revealed that 29 samples were new CPV-2a and 31 samples were new CPV-2b due to amino acids substitution at position 297 (Ser-Ala) (Table-1). The number of positive samples for new CPV-2a and new CPV-2b was nearly equal in 2010. For 2018, 5 new CPV-2a, 19 CPV-2c, and 1 FPV were found, but CPV-2b was not detected (Table-3). In this study, most of the CPV had amino acid substitution at positions 324 and 440. Eighty-three CPV had 324 (Tyr-Ile) substitution due to a T-to-A transversion at nucleotide 970 and an A-to-T transversion at nucleotide 971 with the exception of 1 sample of new CPV-2b in 2010 (CPV-VT 56) (Figure-1). Thirty-two out of 34 new CPV-2a and three out of 31 new CPV-2b (CPV-VT 53, CPV-VT 108, and CPV-VT 148) had 440 (Thr-Ala) substitution due to an A-to-G transition at nucleotide 1318 (Table-1). The new CPV-2a in this study was closely related to CPV-2a from Thailand in 2009 (GQ379045, GQ379046, and GQ379047), and from Uruguay in 2010 and 2011 (JF906788 and KM457139), China in 2015 (MF467224), India in 2015 (KX469433), Singapore in 2014 (KY083098), and Vietnam in 2013 (LC214970) (Figures-2 and 3) due to similar amino acids at positions 267 (Tyr), 324 (Ile), and 440 (Ala). However, two new CPV-2a (CPV-VT 13 and 30) were closely related to new CPV-2a from China in 2008 (FJ435343, FJ435345, GU380304, and GU569936) (Figures-2 and 3) due to similar amino acids at positions 267 (Phe), 324 (Ile), and 440 (Thr). All CPVs in this study had 267 (Phe-Tyr) due to an A-to-T transversion at nucleotide 800 substitution except for the two new CPV-2a samples (CPV-VT 13 and 30) that did not have 440 (Thr-Ala) substitution and one new CPV-2a (CPV-VT 37) that had substitution at position 440. All CPV-2a samples had valine at position 555.

**Table-3:** Number of parvoviruses found in each year.

Year	Type of parvoviruses				

	FPV	CPV2a	CPV2b	CPV2c	Total
2010	0	29	31	0	60
2018	1	5	0	19	25
Total	1	34	31	19	85

CPV=Canine parvovirus, FPV=Feline panleukopenia virus

In contrast to new CPV-2a in this study, all new CPV-2b in this study was distanced from CPV-2b reported previously in Thailand in 2008 (FJ869123 and FJ869124) and Europe in 2005 (FJ005263) (Figures-2 and 3) because of the amino substitution at position 324 (Tyr-Ile). However, three new CPV-2b (CPV-VT 53, CPV-VT 108, and CPV-VT 148) were closely related to the new CPV-2b from China in 2010 (JQ743891) and were also closely related to a cluster of new CPV-2a. These three CPV-2b distanced from the other new CPV-2b in this study (Figures-2 and 3) due to amino substitution at positions 324 (Tyr-Ile) and 440 (Thr-Ala). Nineteen CPV-2c in this study had amino acids substitution at positions 267 (Phe-Tyr), 324 (Tyr-Ile), and 370 (Gln-Arg) due to an A-to-G transition at nucleotide 1109 and a G-to-T transversion at nucleotide 1110. CPV-2c in this study was closely related to CPV-2c from Asia including China in 2014 and 2016 (KP260509, KT162005, KT162016, and KY937641), Indonesia in 2013 (LC216905), and Singapore in 2014 (KY083092) due to these three amino acids substitution (Figures-2 and 3), but distanced from the CPV-2c in Europe and South America. Interestingly, one dog with clinical signs of diarrhea was positive for FPV. The six amino acids (at 80, 103, 232, 323, 564, and 568) out of the seven amino acids which determine the canine or feline host range were similar to the reference FPV; however, there was an amino acid substitution at position 93 (Lys-Asn). According to this amino acid substitution, FPV in this study was in the different clades compared to the reference FPV (Figures-2 and 3).

## Discussion

The number of samples positive for new CPV-2a and 2b circulating in central Thailand in 2010 was approximately equal. This result contrasted with a study in Thailand (data collected between 2003 and 2008) in which the predominant genotype in central Thailand was CPV-2a [11]. Interestingly, the majority CPV genotype in 2018 was CPV-2c, but new CPV-2b was not found. This study showed that CPV-2c had become the predominant genotype during the year 2018 in Thailand. Besides of t he diminishing of CPV-2b, there was only one amino acid difference at position 426 between CPV-2b and CPV-2c but there were two amino acids differences at positions 426 and 440 between CPV-2a and CPV-2c. Thus, the emergence of CPV-2c in Thailand might have derived from the new CPV-2b (2010) that had a mutation at position 426 (Asp-Glu).

From the previous study in Thailand (2003-2009), both CPV-2a and CPV-2b had alanine at amino acid position 297 [11]. According to this finding, CPV-2a and CPV-2b (2003-2009) were new CPV-2a and new CPV-2b. However, most of the CPV in this study had additional amino acid substitution at position 324 (Tyr-Ile), except for 1 CPV-2b (CPV-VT 56). Thus, the new CPV-2b (2010) was different from the new CPV-2b in Thailand (2003-2009) and Europe (2005). The amino acid substitution at residue 324 (Tyr-Ile) was also reported in Brazil [18], China [19], Hungary [20], India [21,22], Italy [23], Nigeria [24], South Korea [25], Taiwan [1,15], and Uruguay [26]. According to the above, amino acid position 324 has been shown to undergo a strong positive selection in the parvovirus of all carnivores [27]. Amino acid position 324 was also adjacent to the critical amino acid position 323 that has been known to control canine cell infection. The changes in the region of the capsid surface around *VP2* residue 300, within a raised region on the shoulder of the three-fold spike of the capsid, have been shown to influence the binding of the virus to the canine TfR [28-30]. This change may influence the interactions with their various host receptors and may be expected to result in an increased host range.

Moreover, most of the current new CPV-2a had alanine at amino acid position 440; however, most of the new CPV-2b still had threonine. Amino acid substitution at position 440 (Thr-Ala) was also reported in the previous studies in China [19], India [21,22], Nigeria [24,31], Pakistan [32], South Africa [33], South Korea [25], and Uruguay [26]. Amino acid position 440 is also important because it is located at the top of the three-fold spike, the main antigenic site of the virus [34,35]. Interestingly, this mutation was not detected in a previous study in Thailand [11]. In the current study, two new CPV-2a (CPV-VT13 and 30) had phenylalanine and threonine at amino acid positions 267 and 440 similar to CPV-2a in 2003-2004 as reported in the previous study in Thailand, but these two strains had isoleucine at amino acid position 324 as seen in CPV-2a in 2008-2009 and new CPV-2a in this study [11]. However, most of the current new CPV-2a in the current study had tyrosine and alanine at positions 267 and 440, as seen in CPV-2a in 2008-2009, respectively. A CPV-2a (CPV-VT37) had phenylalanine and isoleucine at positions 267 and 324 similar to CPV-2a in 2003-2004 but had alanine at amino acid position 440 as seen in CPV-2a in 2008-2009 and new CPV-2a in this study. CPV-VT13, 30, and 37 might represent the transition evolution of the original new CPV-2a to the current new CPV-2a. CPV-2b (CPV-VT56) had similar amino acids at amino acid positions 267, 324, 426, and 440 (phenylalanine, tyrosine, aspartic acid, and threonine, respectively), as reported in the previous study in Thailand [11]. However, new CPV-2b in this study had isoleucine at amino acid 324. Thus, CPV-VT56 might also represent the transition evolution of the original new CPV-2b in the current new CPV-2b.

The CPV-2c in this study was similar to CPV-2c in Asia, such as in China [36], Laos [14], and Taiwan [15,37] due to amino acid substitution at amino acid positions 267 (Tyr), 324 (Ile), and 370 (Arg). CPV-2c in this study differs from CPV-2c in Europe and South America at three amino acid positions (Phe267Tyr, Tyr324Ile, and Gln370Arg). The amino acid substitution at position 440 (Thr-Ala) has been found in Argentina, but this change was not found in this study [12]. Interestingly, FPV was found in a dog that had clinical signs of diarrhea. This FPV sample had an amino acid substitution at position 93 (Lys-Asn). The amino acid at position 93 is important because it is one of the amino acids that determine the canine host range [38]. FPV infection in dogs has also been reported in Pakistan [39]. These findings demonstrated that parvoviruses in Thailand have been dynamically evolving as those in the other part of the world [40,41]. This mutation rate is as high as seen in RNA viruses [41]. The rapid mutation of CPV has resulted in growing concern about the effectiveness of vaccines regarding the new mutant or genotype of CPV. Molecular surveillance of CPV is crucial for the prediction of disease severity and may be important for the development of more effective vaccines or diagnostic tests in the future.

## Conclusion

Two genotypes of CPV (new CPV-2a and CPV-2c) are circulating in Central Thailand and the predominant circulating genotype of CPV has been changed from CPV-2a in the past to CPV-2c at present. Currently, CPV-2b has not been found in Central Thailand. The current new CPV-2a circulating in Thailand has amino acid substitutions at positions 324 (Tyr-Ile) and 440 (Thr-Ala). FPV was found in a dog that had acute diarrhea; however, the importance of this finding remains to be determined. Our results provided additional information on the dynamic evolution of CPV in Thailand, which is following the same evolutionary trend observed in the others part of the world.

## Authors’ Contributions

NI and TS designed the experiment and made DNA extraction, PCR, multiple alignment, and phylogenetic study. NM, SK, KS, and TS were involved in scientific discussion and provided suggestions for the overall work. All authors read and approved the final manuscript.
